# Study on hyperspectral estimation model of soil organic carbon content in the wheat field under different water treatments

**DOI:** 10.1038/s41598-021-98143-0

**Published:** 2021-09-20

**Authors:** Chenbo Yang, Meichen Feng, Lifang Song, Chao Wang, Wude Yang, Yongkai Xie, Binghan Jing, Lujie Xiao, Meijun Zhang, Xiaoyan Song, Muhammad Saleem

**Affiliations:** 1 Agronomy College, Shanxi Agricultural University, Taigu, 030801 Shanxi China; 2 Institute of Geography Science, Taiyuan Normal University, Jinzhong, 030619 Shanxi China

**Keywords:** Ecology, Information technology, Optical spectroscopy

## Abstract

Hyperspectral remote sensing technology can be used to monitor the soil nutrient changes in a rapid, real-time, and non-destructive manner, which is of great significance to promote the development of precision agriculture. In this paper, 225 soil samples were studied. The effects of different water treatments on soil organic carbon (SOC) content, and the relationship between SOC content and spectral reflectance (350–2500 nm) were studied. 17 kinds of preprocessing algorithm were performed on the original spectral (R), and the five allocation ratios of calibration to verification sets were set. Finally, the model was constructed by partial least squares regression (PLSR). The results showed that the effects of water treatment on SOC content were different in different growth stages of winter wheat. Results of correlation analysis showed that the differential transformation can refine the spectral characteristics, and improve the correlation between SOC content and spectral reflectance. Results of model construction showed that the models constructed by second-order differential transformation were not good. But the ratio of standard deviation to the standard prediction error (RPD) values of the models were constructed by simple mathematical transformation (T0–T5) and first-order differential transformation (T6–T11) can reach more than 1.4. The simple mathematical transformation (T0–T2, T4–T5) and the first-order differential transformation (T6–T10) resulted in the highest RPD in mode 5 and mode 2, respectively. Among all the models, the model of T7 in mode 2 reach the highest accuracy with a RPD value of 1.9861. Therefore, it is necessary to consider the data preprocessing algorithm and allocation ratio in the process of constructing the hyperspectral monitoring model of SOC.

## Introduction

Soil organic matter is the material derived from living organisms in the soil, of which 60–80% of the carbon component is called SOC. It is well known that SOC is a vital factor to improve soil fertility, although the content is small relative to the soil^1–3^. However, the previous methods^4^ to obtain SOC content are time-consuming and laborious. Hyperspectral technology can solve this problem. In recent years, with the development of hyperspectral remote sensing technology, due to its characteristics of rapid, real-time, and non-destructive monitoring of targets^5^, it has been become an important technology for many scholars to study the soil properties^6–8^.

The spectral reflectance refers to the ratio of reflected flux to the incident flux at a certain wavelength^9^. Various molecules in the soil absorb and reflect light at different wavelengths to form soil spectral characteristic curve. The visible (350–780 nm) and near-infrared (780–2500 nm) bands have been widely used in agricultural research^10,11^. In the process of quantitative analysis of chemical measurement values and hyperspectral data, analysis result is mainly affected by three aspects: the expression ability of hyperspectral data^12–17^, the data reasonable allocation of calibration to validation sets^18^, and the scientific selection of model construction method^19–23^. The expression ability of hyperspectral data refers to reducing the noise of original spectral data through different spectral transformation algorithms, and improving the correlation between the spectral data and the chemical measurement values^24,25^. The data reasonable allocation of calibration to verification sets will be affected by the amount of data and the degree of dispersion, which is also a segment that is easy to be ignored in the process of model construction^26^. The scientific selection of the modeling method is determined by the quantitative relationship between the chemical measurement values and the spectral data. At present, there are many kinds of methods to construct hyperspectral monitoring model. Some simple linear modeling algorithm include stepwise multiple linear regression (SMLR), partial least squares regression (PLSR), and principal component regression (PCR), etc. Nonlinear modeling algorithm include artificial neural network (ANN), support vector machine (SVM), and random forests (RF)^27–29^, etc. Among them, PLSR is a kind of linear regression model which has been widely used and has a good prediction effect^30–33^.

Previous studies have shown that hyperspectral monitoring has a good ability to predict SOC content^34,35^. PLSR has been used to monitor SOC in many studies, but the results were different. Amin et al.^36^ constructed a hyperspectral monitoring model of SOC content based on PLSR in Azerbaijan, and found that the model constructed after Savitzky-Golay smoothing can reach the highest accuracy with R^2^ and RPD are 0.85 and 2.54, respectively. Yu et al.^37^ constructed a hyperspectral monitoring model of soil organic matter content by using PLSR after preprocessing the spectral data, and the results showed that the model besed on continuous removal (CR) preprocessing had the best accuracy. Ji et al.^18^ used a variety of modeling methods to predict soil organic matter content based on different data allocation ratios, it was found that when the ratios of calibration to validation sets were different, the accuracy of model constructed by different modeling methods was different. But the data allocation ratios used in this study were few, and hyperspectral data were not preprocessed. It can be seen that previous researchers have done some research on data preprocessing and sample allocation ratio in the process of building the SOC model. From the results, it is necessary to consider these two factors in modeling. However, there are few studies considering these two factors at the same time. Based on this, we conducted this study.

According to previous studies, different data preprocessing algorithm, data allocation ratio, and modeling method all have an effect on the accuracy of the model. In this paper, from the perspective of the data allocation ratio, different mathematical transformations of spectral data were carried out, and finally constructs the model by using PLSR. The purposes of this study are: (1) Study the effect of mathematical transformation algorithm and data allocation ratio on the accuracy of hyperspectral monitoring model of SOC content, (2) Find the best spectral preprocessing algorithm and data allocation ratio to predict SOC content.

## Materials and methods

### Experimental design

The experimental field located in the Experiment Station of Shanxi Agricultural University. Each experimental plot is all a square of 9 m^2^, with a total of 15 plots. The water content was controlled according to the percentage of the maximum soil field capacity. For example, when the maximum field capacity of soil is 0.2 g·g^−1^ soil, 80% of the maximum field capacity is 0.16 g·g^−1^ soil. The soil water content was kept at the same level from the returning green stage of winter wheat. There were five irrigation levels: 80%, 60%, 45%, 35%, and 30% of maximum soil field capacity. The experiment was set up in a completely randomized design with three replications. The winter wheat cultivar ‘Zhongmai 175’ was planted. The soil drill was used to collect topsoil of 0–20 cm in critical growth period such as jointing stage, booting stage, heading stage, flowering stage, filling stage, and maturity stage of winter wheat. Use the 5-point sampling method to collect soil samples and mix them for standby. The experiment was carried out for two years, sampling 8 times and 7 times in 2018 and 2019, respectively. The soil texture for the experimental field is calcareous cinnamon soil developed from loess parent material with medium fertility. The average total nitrogen content, the total phosphorus content, and the total potassium content are 0.70 g·kg^−1^, 1.32 g·kg^−1^, and 22.13 g·kg^−1^, respectively. All methods were performed in accordance with Chinese guidelines and legislation.

### Indexes measurement

Before the measurement, the animal, plant residues, and other impurities were removed from samples. After drying in the shade at 25–30 °C for one week, passed through a 0.154 mm sieve. Then measured soil spectral reflectance and SOC content.

#### Soil reflectance measurement

The Field-spec 3 hyperspectral radiometer produced by ASD company in the United States was used for soil spectral measurement. The wavelength acquisition range is 350–2500 nm, among the spectral sampling intervals of 350–1000 nm and 1000–2500 nm are 1.4 nm and 2 nm, respectively, and the spectral resolutions are 3 nm and 10 nm, respectively. Before the measurement, each soil sample with a flat surface was placed in a culture dish. Use the plant probe with its own light source for measurement, and the measurement area is 3.14 cm^2^. Three points were measured for each sample, and each point was measured 10 times. Before each measurement, the whiteboard was used for calibrating.

#### SOC content measurement

SOC content was measured by the Walkley–black method^30^.

### Data processing and analysis

There are 17 common mathematical algorithms were selected to preprocessing the original spectral data of soil, as it showed in Table 1.

**Table 1:** Soil spectral preprocessing mathematical algorithms.

Simple mathematical transformation		First-order differential transformation		Second-order differential transformation	
Code	Algorithm	**Code**	Algorithm	**Code**	Algorithm
T0	*R*	**T6**	*R’*	**T12**	*R’’*
T1	*1/R*	**T7**	*(1/R)’*	**T13**	*(1/R)’’*
T2	*Log R*	**T8**	*(Log R)’*	**T14**	*(Log R)’’*
T3	*1/Log R*	**T9**	*(1/Log R)’*	**T15**	*(1/Log R)’’*
T4	$\surd R$	**T10**	*(*$\surd R$*)’*	**T16**	*(*$\surd R$*)’’*
T5	*1/*$\surd R$	**T11**	*(1/*$\surd R$*)’*	**T17**	*(1/*$\surd R$*)’’*

R represents the original spectrum, the same below.

In this paper, 225 samples were divided into calibration set and verification set by concentration gradient method^38^, including 5 samples allocation ratios (calibration set: verification set): mode 1(2:2), mode 2(3:2), mode 3(4:2), mode 4(5:2), mode 5(6:2).

In this paper, PLSR models were constructed in MATLAB 2010, and leave one out cross-validation method was selected to improve the stability of the model. The coefficient of determination (R^2^), root mean square error (RMSE), and RPD were used to evaluate the accuracy of the model.

This paper also uses View Spec Pro 6.0 to preprocess soil spectral data, Microsoft Excel 2010, Unscrambler 9.7, and SPSS Statistics 20 for data processing, Origin 2016 for mapping.

## Results and analysis

### Change of SOC content

**Figure 1:**
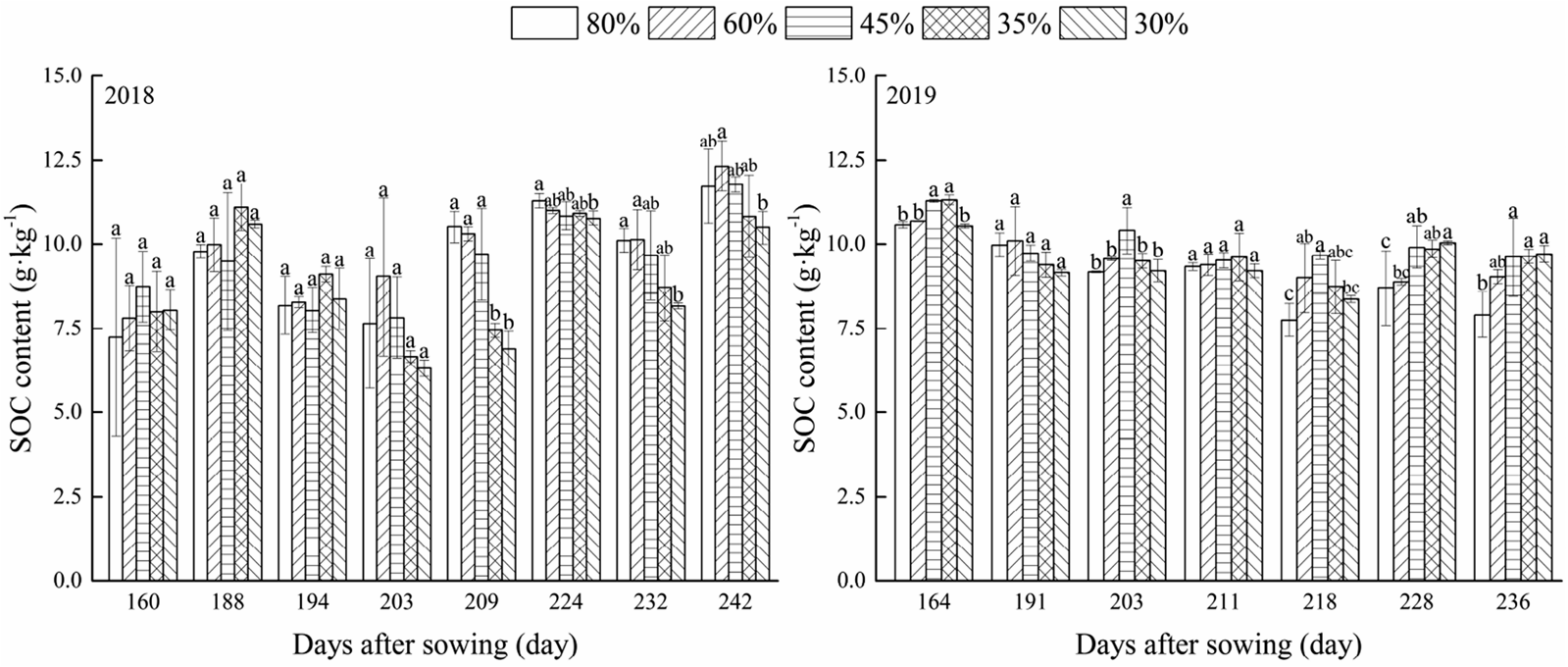
Changes of SOC content under different water treatments.

It can be seen from Fig. 1 that the change trends of SOC content are different with the advance of the winter wheat growth process in the two-year experiment. In 2018, there is an obvious increase–decrease-increase trend, while in 2019 is slowly decreasing to a stable trend. We compare the sampling stages that have close days after sowing in two-year. 160 days and 203 days after sowing in 2018 correspond to 164 days and 203 days after sowing in 2019, respectively. SOC content increased first and then decreased with the aggravation of drought stress, but their significance of difference was different. Although the change trends of 188 days and 194 days after sowing in 2018 and 191 days after sowing in 2019 were different, there was no significant difference in SOC content among the three growth stages. SOC content in 209 days, 224 days, 232 days, and 242 days after sowing in 2018 basically decreased with the aggravation of drought stress, while the change trends of 211 days, 218 days, 228 days, and 236 days after sowing in 2019 were opposite.

### Descriptive statistical analysis

225 samples were analyzed in this paper, it can be seen from Table 2 that the standard deviation of the SOC content was small. The samples have a certain negative skewness, and the data distribution trend was slower than the standard normal distribution, but basically conformed to the normal distribution. In different allocation modes, the minimum and maximum values of the total data set were all assigned to the calibration sets. The average value and standard deviation of the calibration sets and the verification sets were close. This means that the data dispersion was similar. At the same time, the calibration sets and verification sets in the five modes all basically conformed to normal distribution, which can be used for subsequent processing.

**Table 2:** Descriptive statistical analysis of test samples.

	Num	Min (g·kg^−1^)	Max (g·kg^−1^)	Ave	SD	Skewness	Kurtosis
Total	225	4.296	13.013	9.226	1.678	− 0.288	− 0.098
Cal-set of Mode 1	113	4.296	13.013	9.222	1.702	− 0.314	− 0.008
Ver-set of Mode 1	112	4.631	13.009	9.230	1.661	− 0.263	− 0.151
Cal-set of Mode 2	135	4.296	13.013	9.227	1.682	− 0.295	− 0.065
Ver-set of Mode 2	90	4.632	13.009	9.225	1.682	− 0.283	− 0.085
Cal-set of Mode 3	150	4.296	13.013	9.226	1.678	− 0.287	− 0.096
Ver-set of Mode 3	75	4.632	13.009	9.227	1.690	− 0.296	− 0.026
Cal-set of Mode 4	161	4.296	13.013	9.232	1.688	− 0.291	− 0.046
Ver-set of Mode 4	64	5.041	12.721	9.211	1.666	− 0.290	− 0.156
Cal-set of Mode 5	169	4.296	13.013	9.224	1.691	− 0.301	− 0.040
Ver-set of Mode 5	56	5.041	12.829	9.232	1.655	− 0.253	− 0.209

Num, Min, Max, Ave, SD, Cal-set, and Ver-set are number, minimum, maximum, average, standard deviation of samples, calibration set, and verification set, respectively.

### Changes of original spectral reflectance

**Figure 2:**
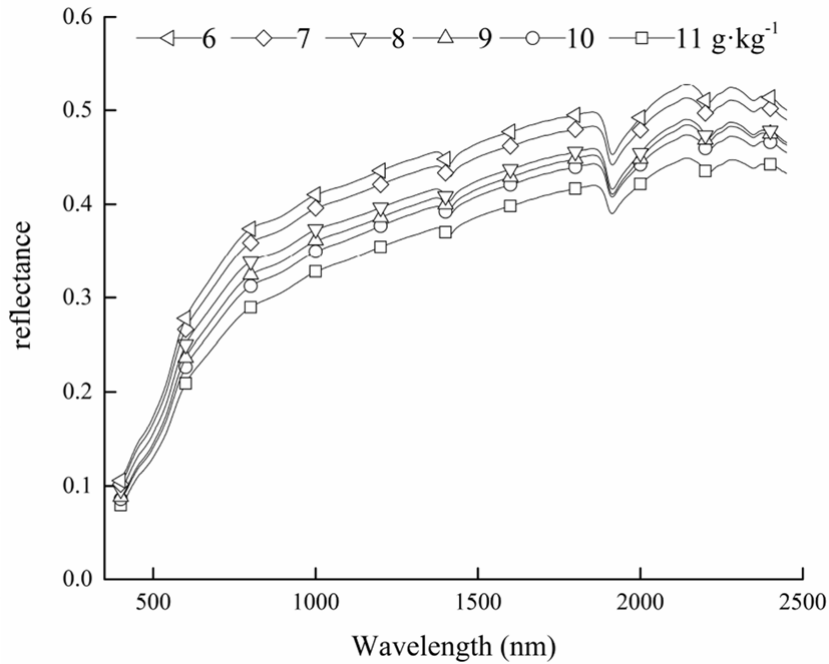
Spectral reflectance changes of soils with different SOC contents.

Since 93% of the 225 samples used in this paper were concentrated in the middle of 6–12 g·kg^−1^, it was necessary to analyse the soil spectral reflectance of SOC content at different levels (6, 7, 8, 9, 10, 11 g·kg^−1^). It can be seen from Fig. 2 that the changing trends of soil spectral reflectance with different SOC content levels were basically consistent. The reflectance increases with the increase of wavelength, and obvious absorption valleys were formed at near 1400 nm, 1900 nm, and 2200 nm. With the increase of SOC content, soil spectral reflectance decreased gradually, but the change range was uneven. The spectral curves of two samples with the SOC content of 6 and 7 g·kg^−1^ were close, and those of 8, 9, and 10 g·kg^−1^ were close to each other.

### Changes of preprocessing spectral reflectance

**Figure 3:**
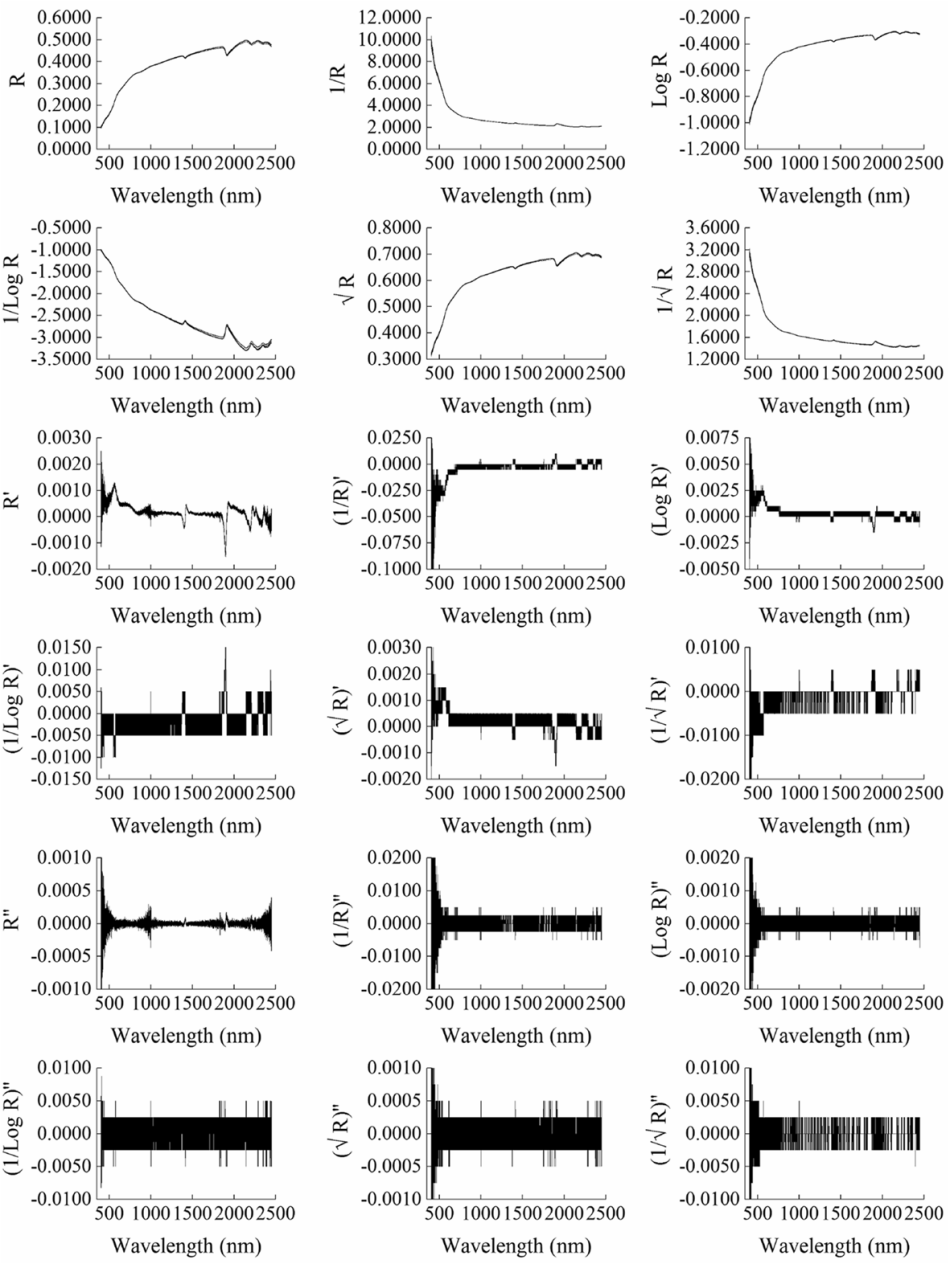
Spectral curves of different preprocessing mathematical algorithms.

In this paper, one sample (SOC content is 9.222 g·kg^−1^) with the closest average content among all samples was taken as an example, and spectral preprocessing was performed according to Table 1. Mapping with directly measured spectral data, as shown in Fig. 3. Figure 3 showed that compared with the original spectral curve (T0), different preprocessing algorithms have different influences. Except for T2 and T4, the changing trends of other spectral preprocessing curves all had an obvious change. Among different types of preprocessing algorithms, the first-order differential transformation and the second-order differential transformation all have an obvious effect on refining spectral characteristics.

### Correlation analysis between SOC content and different preprocessing spectrum

**Figure 4:**
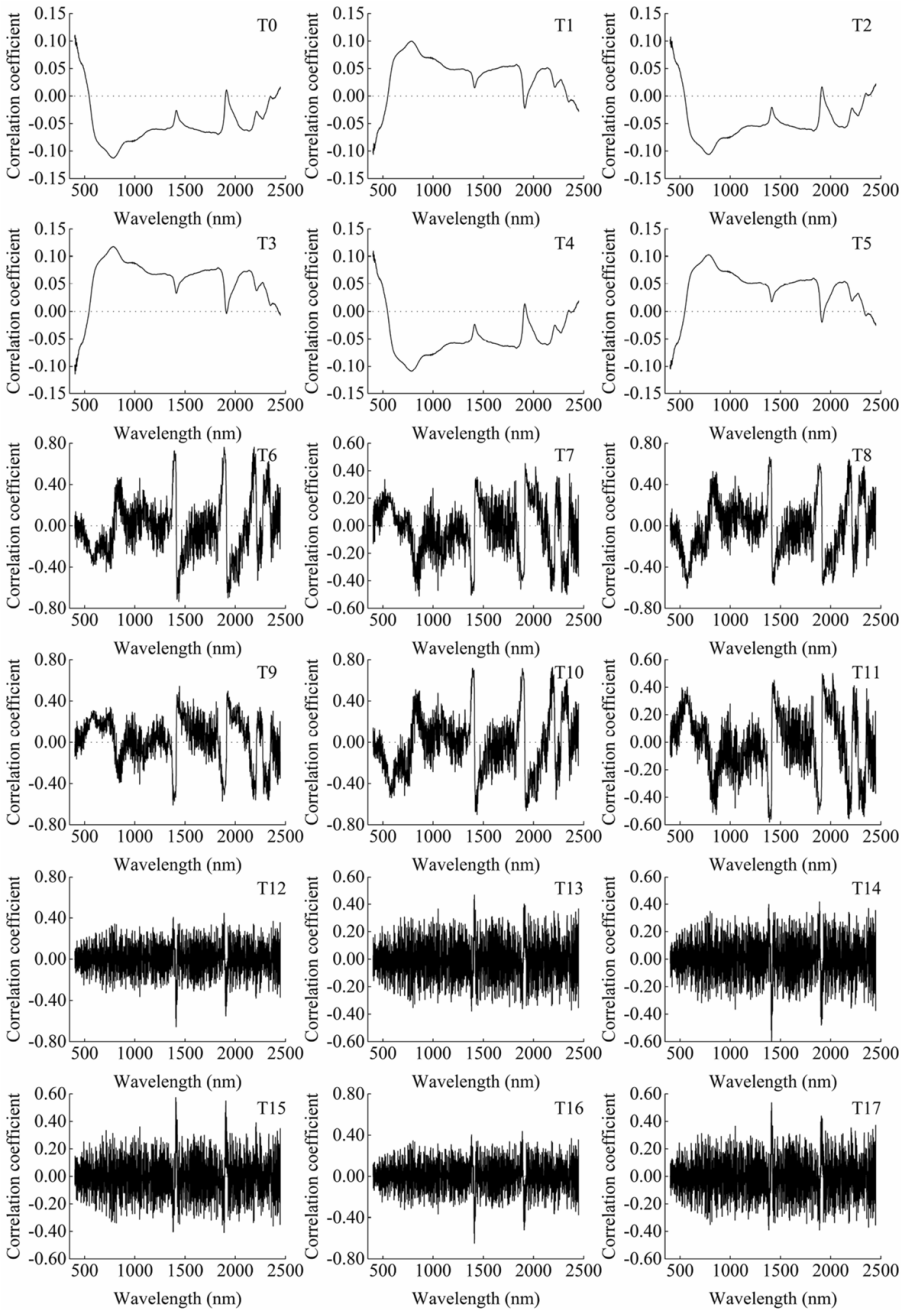
Correlation of different mathematical transformation spectra and SOC content. T0: R; T1: 1/R; T2: Log R; T3: 1/Log R; T4: $\surd$ R; T5: 1/$\surd$ R; T6: R’; T7: (1/R)’; T8: (Log R)’; T9: (1/Log R)’; T10: ($\surd$ R)’; T11: (1/$\surd$ R)’; T12: R’’; T13: (1/R)’’; T14: (Log R)’’; T15: (1/Log R)’’; T16: ($\surd$ R)’’; T17: (1/$\surd$ R)’’, the same below.

It can be seen from Fig. 4 that the correlations between SOC content and the spectral reflectance of T0–T5 were within ± 0.15, while the correlations of T6–T17 were improved. Among them, the correlations of T12–T17 after second-order differential transformation were basically within ± 0.4, and that of T6–T11 after first-order differential transformation were within ± 0.6. In addition, the correlations of T6 and T10 in some wave bands can reach ± 0.7 to ± 0.8. Although differential transformation can improve the correlation between the spectral reflectance and SOC content, the second-order differential transformation has a slight improvement only. However, the first-order differential transformation can improve the correlation greatly. The results showed that the differential transformation may enlarge the spectral characteristics in some wave bands which have a great correlation with SOC content. And the effect of first-order differential transformation was better than second-order differential transformation.

### Construction and validation of PLSR model

**Figure 5:**
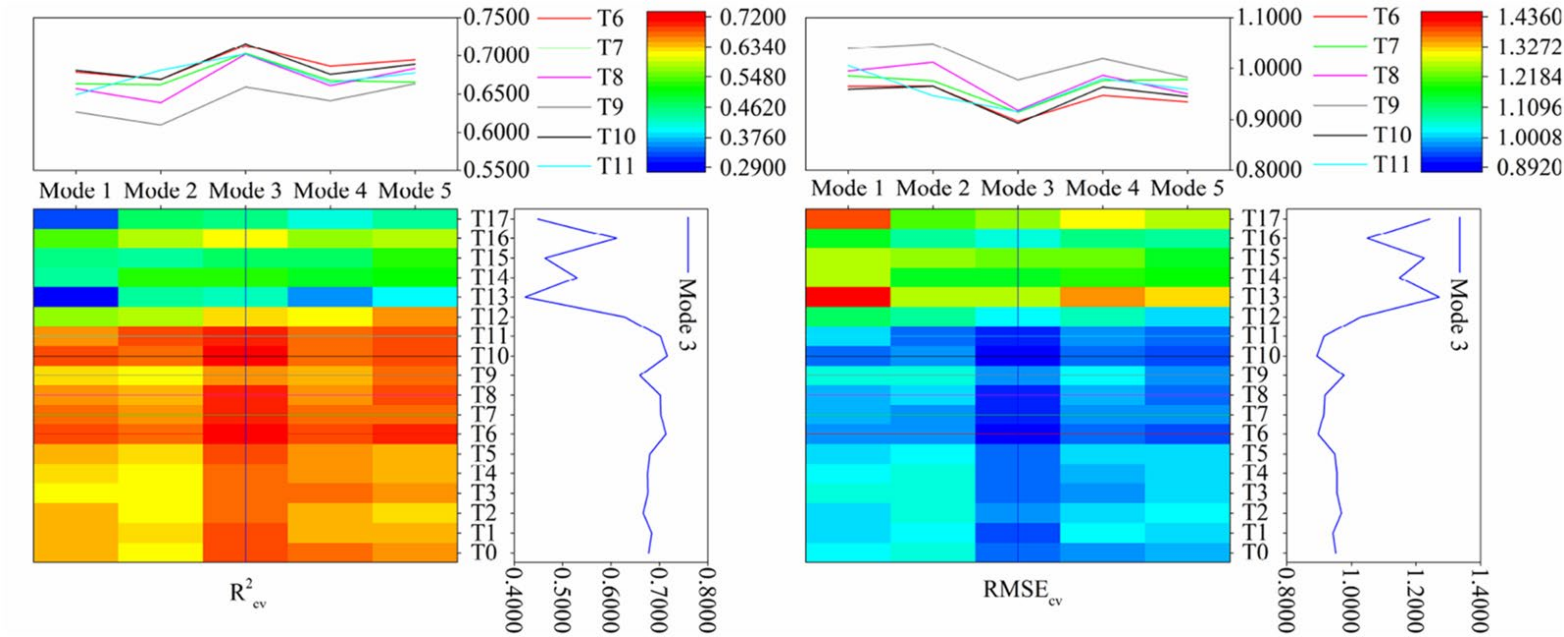
Construction of PLSR models in different modes.

**Figure 6:**
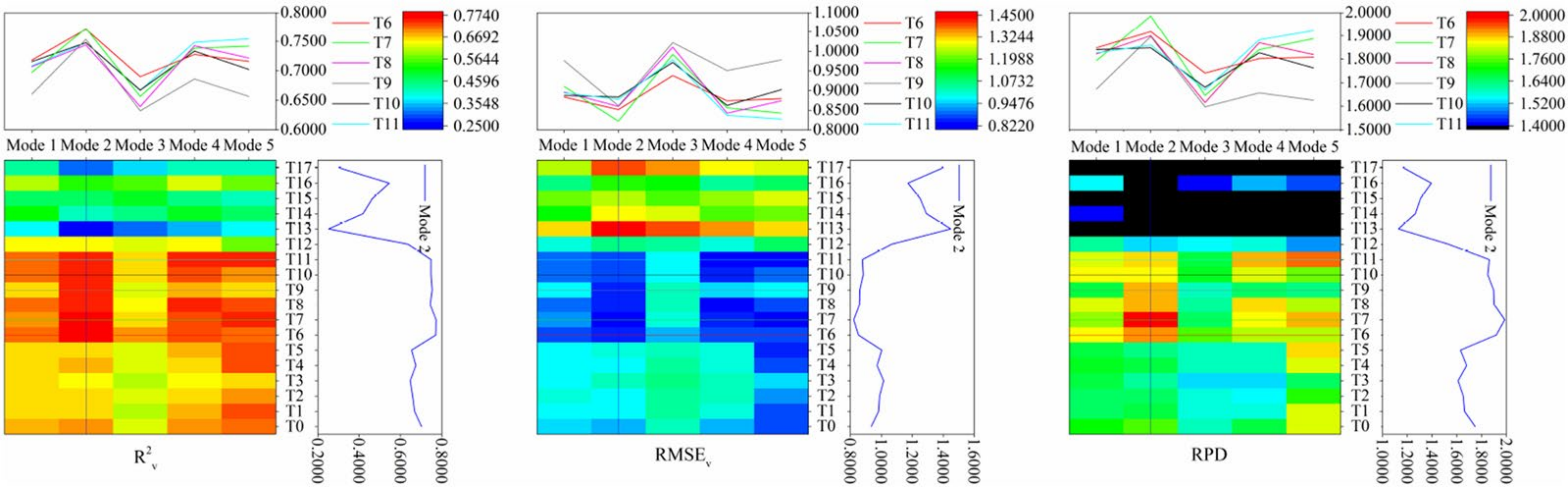
Validation of PLSR models in different modes.

In this study, the PLSR method is used to construct the model, and the model accuracy is shown in Figs. 5 and 6. Figure 5 showed that, compared with the simple mathematical transformation, the accuracy of the models constructed by the first-order differential transformation was increased, but the accuracy of the models constructed by the second-order differential transformation was reduced. Among all the models, the accuracy and stability of T6–T8 and T10–T11 after first-order differential transformation were basically reached the highest in mode 3, and T9 in mode 5 was also higher.

Figure 6 showed that the validation accuracy of the models constructed by the second-order differential transformation was low in all modes. Combined with Fig. 5, it can be seen that the second-order differential transformation was not conducive to construct hyperspectral monitoring models of SOC content by PLSR. The models constructed by simple mathematical transformation and first-order differential transformation achieved the highest verification accuracy in mode 5 and mode 2, respectively. At the same time, the verification accuracy of each model constructed by first-order differential transformation was higher than that of the corresponding simple mathematical transformation. Combined with Fig. 5, it can be seen that the models accuracy can be improved by first-order differential transformation. Different from Fig. 5, the accuracy of the models constructed by simple mathematical transformation and first-order differential transformation reached the lowest in mode 3. It can be seen that it is necessary to consider both the sample allocation ratio and mathematical transformation algorithm when constructing the hyperspectral monitoring model of SOC content. Among all the models constructed in this study, T7 and T11 reached the highest accuracy in mode 2 and mode 5, RPD are 1.9861 and 1.9217, respectively.

## Discussion

From Fig. 1, we can see that the change trends of SOC content with the advance of the winter wheat growth process are different in the 2-year experiment. The change trend of SOC content show an opposite trend with the aggravation of drought stress appears in the later stage of the 2 years experiment. This may be due to the different climatic conditions lead to the difference in the growth of winter wheat in the 2-year experiment. In fact, the growth and development conditions of winter wheat in the first year was better than in the second year. Severe high temperature and drought weather occurred in the experimental plot from March to April of 2019. During this period, the winter wheat is in the stage from returning green to jointing, and the water demand is high. The high temperature leads to water shortage of crops and affects the later growth^39^. In May 2019, the temperature of the experimental site fluctuated greatly, frost disaster occurred in the early stage, and high temperature above 35 °C appeared in the later stage. The extreme temperature further affected the growth of winter wheat^40^.

In this paper, the change of the spectral reflectance of soil with different SOC content have been compared. It was found that the spectral reflectance of soil with different SOC content increased gradually with the increase of wavelength, and obvious absorption valleys were formed near 1400 nm, 1900 nm and 2200 nm. Physical studies show that the spectrum in the visible region is caused by the outer electron transition, while the near-infrared spectrum is mainly affected by molecular vibration, which can reflect the composition and structure of molecules. This is the basic principle of quantitative analysis of target materials by using hyperspectral technology. It ensures that almost no two substances have the same spectral characteristics, but also ensures that the same kind of substances have certain similar spectral characteristics^41^. These absorption valleys are caused by water molecules, OH^-^ and minerals^42^. At the same time, it was also found that the overall value of soil spectral reflectance will decrease with the increase of SOC content, which was consistent with the previous research results^43^. This may be due to the higher content of organic carbon molecules absorbed more light, which may also be one of the principles of quantitative monitoring SOC content with hyperspectral.

Compared with the original spectral curve, 17 mathematical transformations amplify the spectral characteristics to varying degrees. And the effect of differential transformation is the most obvious. Previous researchers generally believe that differential transformation has a good effect in refining spectral features, which is consistent with the results of this paper^44,45^.

In terms of improving the correlation between SOC content and spectral data, simple mathematical transformation (T1–T5) did not significantly improve compared with T0. T2 and T4 were basically the same as T0, while T1, T3, and T5 only changed the positive and negative correlation at the some wavelengths. The first-order and second-order differential transformation improved the correlation between spectral reflectance and SOC content to varying degrees. And the effect of first-order differential transformation was better than second-order differential transformation. At the same time, the results of model construction showed that the RPD value of the models constructed by simple mathematical transformation and first-order differential transformation reached more than 1.4, which were higher than that of the second-order differential transformation. It can be seen, from the mathematical characteristics of the first-order differential, that the spectrum characteristic of SOC is not only limited to some individual wavelengths, but also related to the increase and decrease of some continuous wave bands. At the same time, it is necessary to properly preprocess the spectral data before constructing the hyperspectral monitoring model.

Extracting sensitive band or using full band is a common way to construct hyperspectral quantitative monitoring model of SOC content. Some studies have shown that 435 nm, 500 nm, 965 nm, 1409 nm, 1910 nm, 2170 nm, 2260 nm, and 2380 nm are sensitive bands of SOC^42,46^. Although the model constructed by using sensitive bands can be more concise and efficient, it can be seen from Fig. 4 that the spectral characteristics of SOC may also be related to the change trend of spectral curve. In this case, it is necessary to consider using the full band. PLSR can effectively deal with the complex relationship of high-dimensional data and provide a quantitative model. Therefore, this paper uses PLSR as the model construction method.

In this paper, it was found that the R^2^_cv_ of the models constructed by the second-order differential transformation were mostly below 0.6, and the RMSE_cv_ were higher, the accuracy of model construction was lower than that of simple mathematical transformation and first-order differential transformation. At the same time, the model validation results also showed that the accuracy of models constructed by second-order differential transformation was lower than that of simple mathematical transformation and first-order differential transformation, except for individual models. The accuracy of the models with different sample allocation ratios was different under the same mathematical transformation. Except T3 and T11 reached the highest RPD in mode 1 and mode 5, respectively. Other simple mathematical transformation and first-order differential transformation reached the highest RPD in mode 5 and mode 2, respectively. Generally speaking, among the different mathematical transformation algorithms, the models constructed by the first-order differential transformation have the best accuracy. The model construction and verification accuracy was the best in mode 3 and mode 2, and reached the highest in T10 and T7, respectively.

In this study, R^2^_cv_ and RMSE_cv_ were obtained by cross-validation that were used as the indicators to evaluate the accuracy of model construction. Therefore, with the increase of the number of samples in the calibration set, the more time it takes and the higher the requirement for a computer to construct the model using PLSR. In practice, mode 2 can save more time and computer memory. Therefore, in the process of constructing a hyperspectral monitoring model of SOC content, the first-order differential transformation combined with a sample allocation ratio of 3:2 (calibration set: validation set) is conducive to improve the model accuracy and stability.

## Conclusion

The prediction accuracy of the hyperspectral monitoring model of SOC content is greatly affected by soil spectral preprocessing mathematical algorithms and the ratio of the calibration set to validation set. Different mathematical transformation preprocessing can enlarge or reduce some characteristic information of the original spectral data. Reasonable data allocation ratio can reduce the excessive redundancy of calibration set. But different mathematical transformation algorithms have different requirements for the ratio of the calibration set to verification set. Therefore, in order to improve the accuracy of the model, it is necessary to comprehensively consider the mathematical transformation algorithm and sample allocation ratio.

This study showed that the effects of drought stress on SOC content were different in different growth stages of winter wheat. And the higher the SOC content, the lower the spectral reflectance. The first-order differential transformation and the second-order differential transformation have a greater role in amplifying spectral information. And they can also significantly improve the correlation between SOC content and spectral reflectance, the maximum correlation can reach ± 0.8. The prediction accuracy of the hyperspectral monitoring model of SOC content can be effectively improved by first-order differential transformation and the sample allocation ratio of calibration set: verification set = 3:2. Among them, the model constructed by the first reciprocal transformation and then the first-order differential transformation has the highest accuracy, and the RPD of the verification model is 1.9861.
